# Supplementary material for: Identification and functional analysis of non-coding regulatory small RNA FenSr3 in Bacillus amyloliquefaciens LPB-18
Source: PeerJ. 2023 May 15;11:e15236. doi: 10.7717/peerj.15236 (PMC10194069; doi:10.7717/peerj.15236)
Supplement: Supplemental Information 4 [file peerj-11-15236-s004.zip › KO/CK-vs-T1_map/map04114.html]

KEGG PATHWAY: Oocyte meiosis - Reference pathway


|  |  |
| --- | --- |
| **Oocyte meiosis - Reference pathway** |  |

[
Pathway menu
| Organism menu
| Pathway entry
| Show description
| User data mapping
]

During meiosis, a single round of DNA replication is followed by two rounds of chromosome segregation, called meiosis I and meiosis II. At meiosis I, homologous chromosomes recombine and then segregate to opposite poles, while the sister chromatids segregate from each other at meoisis II. In vertebrates, immature oocytes are arrested at the PI (prophase of meiosis I). The resumption of meiosis is stimulated by progesterone, which carries the oocyte through two consecutive M-phases (MI and MII) to a second arrest at MII. The key activity driving meiotic progression is the MPF (maturation-promoting factor), a heterodimer of CDC2 (cell division cycle 2 kinase) and cyclin B. In PI-arrested oocytes, MPF is initially inactive and is activated by the dual-specificity CDC25C phosphatase as the result of new synthesis of Mos induced by progesterone. MPF activation mediates the transition from the PI arrest to MI. The subsequent decrease in MPF levels, required to exit from MI into interkinesis, is induced by a negative feedback loop, where CDC2 brings about the activation of the APC (anaphase-promoting complex), which mediates destruction of cyclin B. Re-activation of MPF for MII requires re-accumulation of high levels of cyclin B as well as the inactivation of the APC by newly synthesized Emi2 and other components of the CSF (cytostatic factor), such as cyclin E or high levels of Mos. CSF antagonizes the ubiquitin ligase activity of the APC, preventing cyclin B destruction and meiotic exit until fertilization occurs. Fertilization triggers a transient increase in cytosolic free Ca2+, which leads to CSF inactivation and cyclin B destruction through the APC. Then eggs are released from MII into the first embryonic cell cycle.

|  |  |  |
| --- | --- | --- |
| Reference pathway | 184% 150% 122% 100% 82% 67% 55% | 图片下载 |
